# Synergistic effects of <em>Leucaena leucocephala</em>–ginger phytogenic pellet on rumen fermentation, microbial protein synthesis, and enteric methane mitigation in Thai native beef cattle

**DOI:** 10.14202/vetworld.2026.2802-2815

**Published:** 2026-07-10

**Authors:** Parichat Wadjeam, Kampanat Phesatcha, Maharach Matra, Thiwakorn Ampapon, Burarat Phesatcha

**Affiliations:** 1 Department of Applied Biology, Faculty of Sciences and Liberal Arts, Rajamangala University of Technology Isan, Nakhon Ratchasima, Thailand.; 2 Department of Animal Science, Faculty of Agriculture and Technology, Nakhon Phanom University, Nakhon Phanom, Thailand.; 3 Division of Animal Science, Department of Agricultural Technology, Faculty of Technology, Mahasarakham University, Maha Sarakham, Thailand.; 4 Department of Animal Science, Faculty of Agriculture and Technology, Rajamangala University of Technology Isan, Surin Campus, Surin 32000, Thailand.

**Keywords:** beef cattle nutrition, enteric methane mitigation, ginger, Leucaena leucocephala, microbial protein synthesis, phytogenic feed additive, rumen fermentation, Thai native beef cattle

## Abstract

**Background and Aim:**

Enteric methane (CH_4_) emissions from ruminants contribute substantially to greenhouse gas accumulation and represent an energy loss that reduces feed efficiency. Phytogenic feed additives rich in bioactive compounds have attracted attention as sustainable alternatives to manipulate rumen fermentation. This study aimed to develop and evaluate a novel *Leucaena leucocephala*–ginger phytogenic pellet (LGP) and determine its effects on nutrient utilization, rumen fermentation, microbial protein synthesis, and CH_4_ mitigation in Thai native beef cattle.

**Materials and Methods:**

Four female Thai native beef cattle (230 ± 10 kg) were assigned to a 4 × 4 Latin square design. Animals received a basal diet consisting of concentrate at 1.0% of body weight and rice straw, supplemented with LGP at 0, 50, 100, or 150 g/head/day. Each experimental period lasted 21 days, including 14 days of adaptation and 7 days of sampling. Feed intake, nutrient digestibility, rumen fermentation characteristics, blood metabolites, microbial populations, and microbial protein synthesis were evaluated.

**Results:**

LGP supplementation did not affect feed intake, ruminal pH, ammonia nitrogen concentration, or blood urea nitrogen levels (p > 0.05). However, supplementation improved dry matter digestibility, increasing from 55.4% to 60.0%, and neutral detergent fiber digestibility, increasing from 60.1% to 67.6% (p < 0.05). The highest supplementation level (150 g/head/day) increased ruminal propionate concentration to 28.8 mol/100 mol and enhanced bacterial populations and microbial protein synthesis, reaching 68.1 g N/day (p < 0.05). Protozoal counts declined to 4.8 × 10⁶ cells/mL, accompanied by a reduction in estimated CH_4_ production to 23.7 mM/L (p < 0.05). No adverse health effects or clinical signs associated with mimosine toxicity were observed throughout the experiment.

**Conclusion:**

Supplementation with LGP at 150 g/head/day effectively improved rumen fermentation efficiency, enhanced fiber utilization and microbial protein synthesis, increased propionate production, and reduced protozoal populations and enteric CH_4_ formation. These findings demonstrate the synergistic potential of *L. leucocephala* and ginger as a practical phytogenic feed additive to improve rumen function and promote environmentally sustainable beef production under tropical conditions. Further long-term studies involving direct CH_4_ measurements and production performance evaluations are warranted.

## INTRODUCTION

Emissions of greenhouse gases (GHGs), particularly carbon dioxide (CO_2_) and methane (CH_4_), from livestock production are major contributors to global warming. Increasing demand for livestock products further exacerbates the accumulation of GHGs in the atmosphere. Reducing enteric CH_4_ production in ruminants is therefore an important strategy for improving environmental sustainability and feed utilization efficiency. However, the high cost and limited availability of concentrate feeds remain major constraints for animal production systems, prompting nutritionists to explore affordable and sustainable alternatives. Numerous plant species contain nutrients and bioactive compounds that enhance animal health, and several plant-derived compounds have shown considerable promise as natural feed additives [1]. In tropical regions, leguminous plants are widely utilized to improve the availability and nutritional quality of ruminant diets. Forages, shrubs, legumes, cereals, and grains are rich sources of phytogenic compounds, particularly condensed tannins (CT) and saponins (SP), which have attracted considerable attention as natural rumen modifiers capable of improving feed efficiency and reducing CH_4_ emissions [2]. Depending on their concentration, tannins may exert either antinutritional or beneficial effects, contributing to improved animal productivity and reduced CH_4_ formation. Moreover, the leaves of leguminous trees and shrubs represent inexpensive and readily available sources of dietary protein.

*Leucaena leucocephala*, commonly known as leucaena, has attracted considerable interest as a ruminant feed because of its high crude protein (CP) content, rapid ruminal degradability, and capacity to enhance rumen microbial activity [3]. Recent evaluations have demonstrated favorable *in vitro* rumen fermentation kinetics, indicating that the leaves and stems of *Leucaena* are highly suitable protein supplements for ruminants [4]. Furthermore, several *Leucaena* species have been reported to positively modify fermentation pathways and increase post-ruminal protein supply without adversely affecting total gas production [5]. *Leucaena* contains approximately 31.1% CP, 5.6% crude fat, 94.8% dry matter (DM), 13.2% crude fiber, 4.5% ash, and 1.89% CT [6]. Variations among subspecies and cultivars influence CT concentration, underscoring *Leucaena*'s value as a fodder legume for its high palatability and abundance of protein, amino acids, minerals, and fiber. The ability of *Leucaena* to reduce enteric CH_4_ production is largely attributed to CT, which forms complexes with proteins and polysaccharides, thereby decreasing nutrient degradation in the rumen. In addition, *Leucaena* leaves provide substantial amounts of essential amino acids such as isoleucine and leucine, as well as limiting amino acids such as lysine and methionine, compared with other multipurpose tree species [7]. According to Montoya-Flores* et al.* [8], supplementation with dried *L. leucocephala* leaves at levels up to 12% DM improved digestible CP and reduced daily CH_4_ emissions without negatively affecting DM intake, fermentation characteristics, or rumen microbial populations in crossbred heifers. Similarly, Aoetpah *et al.* [9] reported that Timor Bali cattle fed a Cipelang grass-based diet containing 56% leucaena leaf meal exhibited improved DM intake, nutrient digestibility, and average daily gain. Consequently, natural feed additives have attracted increasing attention as a strategy for enhancing feed utilization and animal performance.

Medicinal plants containing bioactive secondary metabolites with antimicrobial properties have been widely used to improve productivity in pigs, poultry, ruminants, and aquaculture species. Among these, ginger (*Zingiber officinale*) has recently emerged as a potent modulator of the rumen ecosystem and has demonstrated a significant capacity for CH_4_ mitigation under *in vitro* conditions [10]. These findings are consistent with the broader use of phytogenic additives to alter rumen fermentation patterns and reduce GHG emissions [2]. Ginger powder contains approximately 60%–70% carbohydrates, 9% protein, 3%–8% fiber, 2%–6% proteases, 3%–6% lipids, and 1%–3% volatile compounds, including gingerol, zingiberene, zingiberol, shogaol, terpenes, oleoresin, and zingerone, in addition to vitamins A, C, and B3, phenols, and flavonoids [11]. Phytobiotics and phytochemicals derived from ginger enhance nutrient digestibility, growth rate, feed conversion efficiency, and feed palatability. Moreover, ginger promotes animal growth through its antimicrobial activity against pathogenic microorganisms, thereby improving digestive efficiency [12]. Increased salivary secretion and stimulation of digestive enzymes induced by ginger also promote the proliferation of cellulolytic bacteria. Al-Dain and Jarjeis [13] observed that supplementation with 75 or 150 g of ginger root powder/head/day improved feed intake and milk production in dairy cows. Thus, ginger has been extensively investigated as a phytogenic feed additive because of its beneficial effects on animal health and productivity.

Although numerous studies have documented the individual effects of *L. leucocephala* and ginger, a critical knowledge gap remains. Previous studies have primarily focused on the use of *Leucaena* as a source of CT to reduce CH_4_ emissions and enhance bypass protein, whereas the effects of ginger have largely been investigated independently due to its antimicrobial and fermentation-modulating properties. Moreover, most studies evaluating ginger have been conducted *in vitro*, whereas *in vivo* studies assessing its effects in ruminants remain limited. To date, no study has strategically integrated these two phytogenic resources into a practical pelleted formulation intended for beef cattle. Consequently, the potential synergistic interactions among CT from *Leucaena*, flavonoids, and essential oils from ginger on nutrient utilization, rumen fermentation, microbial population dynamics, microbial protein synthesis, and CH_4_ mitigation have not been comprehensively investigated. In addition, information regarding the efficacy of such a combination in animals maintained on low-quality rice straw-based diets is lacking. Despite the excellent nutritional profile of *L. leucocephala*, its utilization is constrained by the presence of mimosine, a toxic non-protein amino acid that may constitute 2%–10% of leaf DM and can adversely affect growth and thyroid function at excessive intake levels. Nevertheless, previous evidence suggests that appropriate processing methods, including drying and pelleting, together with controlled inclusion levels, can effectively minimize these risks.

Considering the complementary biological activities of *L. leucocephala* and ginger, this study hypothesized that co-pelleting *Leucaena* leaves and ginger powder at a 75:15 ratio would generate synergistic effects that improve nutrient degradability, suppress protozoal populations, enhance microbial efficiency, and mitigate enteric CH_4_ production without compromising animal health. To ensure safety, the maximum supplementation level was limited to 150 g/head/day, corresponding to approximately 112.5 g of *Leucaena* leaves, thereby allowing the formulation to function as a phytogenic modulator rather than a major protein source. Therefore, the objective of this study was to develop and evaluate a novel *Leucaena*-ginger phytogenic pellet (LGP) and to determine its effects on nutrient utilization, rumen fermentation characteristics, microbial protein synthesis, microbial populations, and enteric CH_4_ production in Thai native beef cattle. Furthermore, the study aimed to assess the feasibility of this phytogenic formulation as a practical and environmentally sustainable feed additive for tropical beef production systems.

## MATERIALS AND METHODS

### Ethical approval

The study was approved by the Animal Care and Use Committee of Rajamangala University of Technology Isan, Thailand (approval no. 01-67-002). According to the National Research Council of Thailand Guidelines for Ethics of Animal Experimentation, approval was required for rumen fluid collection because the primary objective of this study involved the laboratory evaluation of ruminant feeds. All procedures were conducted in accordance with the applicable regulations and guidelines. The study adhered to the Animal Research: Reporting of *in vivo* Experiments 2.0 guidelines. Throughout the experimental period, all animals were monitored daily for clinical signs of mimosine toxicity and adverse health effects, including alopecia, excessive salivation, lethargy, and abnormal weight loss. No adverse clinical signs were observed during the study.

### Study period and location

The experiment was conducted during the rainy season in August 2024 in Roi Et Province, Thailand. Fresh young leaves of *L. leucocephala* and rhizomes of *Z. officinale* were harvested locally at approximately 45 days of age. The feeding trial and laboratory analyses were performed under tropical conditions in Thailand.

### Study design

Four female Thai native beef cattle with an initial body weight (BW) of 230 ± 10 kg and approximately 2 years of age were randomly assigned to four dietary treatments according to a 4 × 4 Latin square design. The treatments consisted of a control group without LGP supplementation and groups receiving LGP at 50, 100, and 150 g/head/day.

The concentrate allowance was provided at 1.0% of BW on a DM basis. To ensure accurate feeding rates throughout the study, the daily concentrate allocation for each animal was recalculated according to individual BW measured at the beginning and end of each experimental period. Because of the short 21-day duration of each period, average daily gain was not evaluated as a primary parameter; however, no significant BW loss was observed.

Animals received LGP according to their assigned treatments and were fed two equal portions of locally sourced rice straw (*Oryza sativa* L.) at 07:00 and 16:00 h daily. The basal diet was formulated to meet the maintenance and growth requirements of Thai native beef cattle. The chemical compositions of the concentrate mixture, rice straw, and LGP are presented in Table 1. Based on the daily intake proportions, the nutrient composition of the total diet provided sufficient energy and protein to meet the maintenance requirements of the experimental animals.

Each experimental period lasted 21 days, comprising 14 days for dietary adaptation and 7 days for sample collection and nutrient digestibility measurements. During the experiment, the cattle were housed individually in well-ventilated stalls measuring approximately 2 × 3 m, with concrete floors covered with rubber mats, under natural daylight and ventilation. Clean drinking water was available *ad libitum* throughout the experiment.

Before the start of the trial, all animals underwent a comprehensive health examination, including routine vaccination against Foot and Mouth Disease and treatment for internal and external parasites with ivermectin to ensure optimal health.

The chemical compositions and nutrient contents of the experimental diets and LGP are listed in Table 1.

**Table 1:** Feed ingredients and chemical compositions of the concentrate mixture, rice straw, and Leucaena leucocephala–ginger phytogenic pellet used in the experiment.

**Items**	**Concentrate**	**Rice straw**	**LGP**
Feed ingredients (% as fed)			
Cassava pulp	25.0	–	–
Cassava chip	21.5	–	–
Palm meal	34.0	–	–
Rice bran	14.5	–	–
Soybean meal	0.5	–	–
Urea	1.5	–	–
Sulfur	1.0	–	–
Mineral mix	1.0	–	–
Salt	1.0	–	–
Leucaena leaves meal	–	–	75.0
Ginger powder	–	–	15.0
Cassava chip	–	–	9.0
Molasses	–	–	1.0
Chemical composition (%)			
Dry matter (DM)	92.5	93.0	89.5
Organic matter	92.7	91.5	85.2
Ash	7.3	8.5	14.8
Crude protein	14.7	2.2	24.6
Neutral detergent fiber	28.3	75.5	32.5
Acid detergent fiber	15.1	47.4	25.2
Condensed tannins	–	–	6.8
Flavonoids	–	–	2.4

LGP = Pellet from *Leucaena leucocephala* leaves mixed with ginger powder.

### Preparation of phytogenic pellets

Fresh young leaves of *L. leucocephala* and rhizomes of *Z. officinale* were harvested locally in Roi Et Province, Thailand. Following collection, the leaves and chopped ginger rhizomes were dried in a hot-air oven at 60°C for 48 h until a constant weight was achieved. The dried materials were then ground using a Cyclotec Mill (Tecator, Hoganas, Sweden) and passed through a 1-mm screen.

Pellets were prepared by combining 75% *L. leucocephala* leaves, 15% ginger powder, 9% cassava chips, and 1% molasses using a pellet machine. This ratio of active phytogenic ingredients was selected to provide an optimal supply of CT and essential oils to effectively modulate rumen fermentation without impairing microbial nutrient degradability. Following pellet formation, pellets measuring approximately 6-8 mm in diameter were sun-dried for 2-3 days to reduce moisture content and ensure stability during storage and feeding.

Although mimosine and SP concentrations were not quantified because of laboratory limitations, previous studies have demonstrated that the combination of sun drying, oven drying at 60°C, and heat generated during pellet production substantially reduces mimosine concentrations to safe levels. Cassava chips and molasses were incorporated primarily as pellet binders and highly fermentable energy sources, and their nutrient contributions were inherently included in the proximate composition of the final pellets (Table 1). To maintain stability and prolong shelf life, the pellets were stored in sealed moisture-proof containers at room temperature throughout the feeding trial.

### Sample collection and chemical analyses

Feeds were weighed daily to determine the intake of concentrate, rice straw, and LGP for each dietary treatment. Feed, fecal, and urine samples were collected during the last 7 days of each experimental period. Following oven drying, samples were ground to pass through a 1-mm screen and analyzed for DM, ash, and CP according to Association of Official Analytical Chemists procedures [14]. Acid-insoluble ash was determined according to Van Keulen and Young [15]. Acid detergent fiber (ADF) and neutral detergent fiber contents were analyzed according to the method of Van Soest *et al.* [16].

The concentrations of CT and flavonoids in LGP were determined using the Folin-Ciocalteu reagent by measuring absorbance at 765 nm [17].

Spot urine samples were obtained by manual stimulation of the vulva to induce urination, whereas fecal samples were collected directly from the rectum. Samples were collected at 0 and 4 h after the morning feeding. Total daily urine output was estimated from creatinine concentration, assuming a constant creatinine excretion rate of 0.88 mmol/kg BW^0.75. Microbial purine absorption was estimated from the excretion of purine derivatives. Allantoin and creatinine concentrations were measured by high-performance liquid chromatography, and microbial nitrogen supply was calculated using the equations, assumptions, and recovery factors described by Chen and Gomes [18].

Rumen fluid and jugular blood samples were collected at 0 and 4 h after feeding. To minimize stress and discomfort, rumen fluid was collected using a soft flexible stomach tube connected to a vacuum pump, with the sample volume limited to 200 mL. Immediately after collection, ruminal temperature and pH were measured using a handheld pH meter (HI8424 Microcomputer; Hanna Instruments, Woonsocket, RI, USA).

Rumen fluid was filtered through four layers of cheesecloth and divided into two portions. In the first portion, NH_3_-N and VFA concentrations were determined by mixing 45 mL of rumen fluid with 5 mL of 1 M H_2_SO_4_ followed by centrifugation at 1,600 × *g* for 15 min. The NH_3_-N concentration was determined by the micro-Kjeldahl method [14], whereas VFA concentrations were analyzed using high-performance liquid chromatography [19].

CH_4_ production was estimated from VFA concentrations according to Moss *et al.* [20] using the following equation:

CH_4_ production (mol/100 mol total VFA) = 0.45 (acetate) − 0.275 (propionate) + 0.40 (butyrate).

For microbial enumeration, 1 mL of filtered rumen fluid was immediately transferred into 9 mL of 10% formalin solution to preserve microbial cells. Bacterial and protozoal populations were counted directly using a hemocytometer under a phase-contrast microscope, as described by Galyean [21]. To ensure accuracy and reliability, microscopic counts were performed in duplicate, and at least 10 microscopic fields were examined for each sample. To ensure precision and reproducibility, all chemical and microbial analyses were conducted in triplicate.

Blood samples (5 mL) were collected from the jugular vein at 0 and 4 h after feeding, placed in tubes containing ethylenediaminetetraacetic acid, and analyzed for blood urea nitrogen (BUN) according to Crocker [22].

### Statistical analysis

Before statistical analysis, the assumptions of analysis of variance were evaluated. Residual normality was assessed using the Shapiro-Wilk test, and homogeneity of variances was examined to verify the suitability of the statistical model. The use of four animals in a 4 × 4 Latin square design represents standard practice for *in vivo* rumen metabolism studies and balances statistical power with the ethical principle of minimizing animal use.

Potential outliers were evaluated using studentized residuals, and no observations exceeded the ±3 standard deviation threshold. Therefore, all observations were retained for the final analysis.

Data were analyzed using the MIXED procedure in SAS version 9.4 (SAS Institute Inc., Cary, NC, USA) with a variance-covariance structure based on a 4 × 4 Latin square design. The statistical model was:

### Yijk = μ + Ai + Pj + Tk + eijk

where Yijk represents the observation, μ is the overall mean, Ai is the random effect of animal (i = 1, 2, 3, and 4), Pj is the random effect of experimental period (j = 1, 2, 3, and 4), Tk is the fixed effect of treatment (k = 1, 2, 3, and 4), and eijk is the residual error.

Differences among treatment means were determined using Tukey's multiple comparison procedure [23], and significance was declared at p < 0.05. Orthogonal polynomial contrasts were used to evaluate linear and quadratic treatment responses.

## RESULTS

### Chemical composition of experimental feeds

The feed ingredients and chemical compositions of the concentrate mixture, rice straw, and LGP used in the experiment are presented in Table 1. As expected for standard basal diets, the concentrate and rice straw contained 14.7% and 2.2% CP, respectively. Notably, the formulated LGP exhibited a favorable nutritional profile, containing 24.6% CP, 32.5% neutral detergent fiber, and 25.2% ADF. In addition, LGP served as a substantial source of bioactive compounds, providing 6.8% CT and 2.4% flavonoids. *L. leucocephala* is a widely distributed leguminous species in tropical regions with a high protein content and is commonly used as a feed resource for ruminants.

### Feed intake and nutrient digestibility

The effects of dietary treatments on feed intake and nutrient digestibility are presented in Table 2. Supplementation with LGP had no effect on rice straw intake, concentrate intake, or total feed intake among treatment groups (p > 0.05). However, increasing LGP levels (p < 0.05) increased the digestibility of DM and neutral detergent fiber. In contrast, the digestibility of OM, CP, and ADF was not affected by LGP supplementation (p > 0.05).

When expressed as a percentage of BW, total daily DM intake ranged from approximately 2.5% to 2.7% across treatments, which is within the normal range for beef cattle. Feed intake is influenced by the chemical composition and physical characteristics of the diet, which in turn affect animal productivity. The absence of significant differences in feed intake among treatments indicates that LGP supplementation did not adversely affect feed consumption.

**Table 2:** Effects of phytogenic pellets derived from Leucaena leucocephala and ginger on voluntary feed intake and nutrient digestibility in beef cattle.

**Items**	**0**	**50**	**100**	**150**	**SEM**	**Linear**	**Quadratic**	**Cubic**
Rice straw intake, kg DM/day	3.6	3.5	3.8	3.8	0.18	0.43	0.52	0.90
Concentrate intake, kg DM/day	2.1	2.1	2.2	2.3	0.06	0.42	0.18	0.78
								
LGP, kg DM/day	0	0.05	0.10	0.15	–	–	–	–

Total feed intake, kg DM/day	5.7	5.7	6.1	6.2	0.18	0.30	0.84	0.83
Apparent digestibility (%)								
DM	55.4ᵃ	57.4ᵇ	58.9ᵇ	60.0ᶜ	3.46	0.02	0.80	0.95
Organic matter	61.6	64.3	65.0	68.3	3.52	0.23	0.92	0.77
Crude protein	52.2	54.0	57.1	58.5	2.95	0.07	0.81	0.91
Neutral detergent fiber	60.1ᵃ	62.5ᵇ	64.0ᶜ	67.6ᵈ	2.01	0.03	0.80	0.74
Acid detergent fiber	45.2	46.7	48.2	48.8	1.11	0.14	0.73	0.84

^a–^^d^Means within a row with different superscripts differ significantly (p < 0.05). LGP = Pellet from *Leucaena leucocephala* leaves mixed with ginger powder;

SEM = Standard error of the mean; DM = Dry matter.

### Rumen fermentation characteristics, blood metabolites, and microbial populations

The effects of dietary treatments on rumen fermentation characteristics, blood metabolites, and microbial populations are presented in Table 3. Supplementation with LGP had no effect on ruminal temperature, pH, or blood urea nitrogen (BUN) concentration (p > 0.05). Rumen fermentation, microbial growth, and microbial activity were maintained within the optimal pH range of 6.7-6.8. Increasing the level of LGP supplementation did not significantly affect NH_3_-N concentrations, which remained within the normal range of 13.7-18.5 mg/dL. BUN concentrations were closely associated with ruminal NH_3_-N concentrations, although no significant differences in BUN concentrations were observed among treatments.

Increasing LGP supplementation levels resulted in higher propionic acid concentrations and lower estimated CH_4_ production (p < 0.05), particularly at 150 g/head/day. However, total VFA concentration, acetic acid concentration, butyric acid concentration, and the acetic acid-to-propionic acid ratio did not differ significantly among treatment groups (p > 0.05).

Furthermore, increasing levels of LGP supplementation increased bacterial populations (p < 0.05) and decreased protozoal populations (p < 0.05), as shown in Table 3.

**Table 3:** Effects of phytogenic pellets derived from Leucaena leucocephala and ginger on rumen fermentation characteristics, blood urea nitrogen, and microbial populations in beef cattle.

**Items**	**0**	**50**	**100**	**150**	**SEM**	**Linear**	**Quadratic**	**Cubic**
Ruminal pH	6.7	6.7	6.7	6.8	0.12	0.28	0.30	0.05
Ruminal temperature (°C)	37.5	38.1	38.0	38.3	0.25	0.07	0.16	0.54
NH_3_-N concentration (mg/dL)	13.7	16.1	17.2	18.5	0.96	0.93	0.39	0.58
Blood urea nitrogen (mg/dL)	10.3	10.4	11.3	12.0	0.59	0.89	0.75	0.96
Total VFA (mmol/L)	111.0	109.1	108.3	106.9	3.28	0.40	0.93	0.91
VFA (mol/100 mol)								
Acetic acid (C2)	68.8	67.3	66.5	63.6	2.05	0.12	0.74	0.76
Propionic acid (C3)	21.2ᵃ	24.5ᵇ	25.3ᵇ	28.8ᶜ	1.93	0.04	0.93	0.57
Butyric acid (C4)	10.0	8.3	8.2	7.5	1.60	0.34	0.75	0.75
Acetic acid/propionic acid ratio	3.3	2.8	2.8	2.3	1.22	0.07	0.91	0.36
Methane production¹ (mol/100 mol VFA)	29.1ᵃ	26.9ᵇ	26.3ᵇ	23.7ᶜ	1.38	0.02	0.91	0.57
Rumen microbial population (cells/mL)								
Bacteria (×10⁹)	6.3ᵃ	7.2ᵇ	8.3ᶜ	8.7ᶜ	0.36	0.01	0.63	0.83
Protozoa (×10⁶)	8.4ᵃ	6.9ᵇ	5.6ᶜ	4.8ᵈ	0.45	0.03	0.83	0.96

¹Methane production = 0.45(C2) − 0.275(C3) + 0.4(C4), calculated according to Moss *et al.* [20].

^a–^^d^Means within a row with different superscripts differ significantly (p < 0.05).

LGP = Pellet from *Leucaena leucocephala* leaves mixed with ginger powder; SEM = Standard error of the mean, VFA = Volatile fatty acids

### Microbial protein synthesis

The effects of dietary treatments on microbial protein synthesis are presented in Table 4. Supplementation with LGP had no significant effect on absorbed or excreted urinary purine derivatives (p > 0.05). However, microbial protein synthesis and efficiency of microbial nitrogen synthesis increased significantly (p < 0.05) when cattle received 150 g/head/day of LGP.

**Table 4:** Effects of phytogenic pellets derived from Leucaena leucocephala and ginger on urinary purine derivatives and microbial protein synthesis in beef cattle.

**Items**	**0**	**50**	**100**	**150**	**SEM**	**Linear**	**Quadratic**	**Cubic**
Urinary purine derivatives (mmol/day)								
Allantoin excretion	31.5	34.0	36.5	37.3	4.46	0.76	0.14	0.45
Allantoin absorption	90.9	92.6	97.2	98.7	4.40	0.12	0.23	0.26
Microbial protein supply (g N/day)	60.3ᵃ	64.3ᵇ	67.9ᶜ	68.1ᶜ	4.45	0.04	0.16	0.22
Efficiency of microbial nitrogen synthesis (g/kg organic matter digested in the rumen)¹	28.5ᵃ	31.4ᵇ	32.9ᵇ	35.5ᶜ	0.61	0.02	0.28	0.47

¹Organic matter digested in the rumen.

^a–^^c^Means within a row with different superscripts differ significantly (p < 0.05).

LGP = Pellet from *Leucaena leucocephala* leaves mixed with ginger powder; SEM = Standard error of the mean.

The efficiency of microbial nitrogen synthesis and microbial nitrogen supply was calculated from urinary excretion of purine derivatives, using the equations and assumptions described by Chen and Gomes [18].

## DISCUSSION

### Chemical composition of experimental feeds

The high CP content observed in LGP was primarily attributed to the inclusion of *L. leucocephala*, a widely available leguminous species in tropical regions that is commonly used as a high-quality protein feed for ruminants. This finding is consistent with Melesse *et al.* [7], who reported that *L. leucocephala* leaves contain high levels of CP (24.5%), total phenols (6.5%), and CT (2.3%), and provide an excellent supply of essential amino acids compared with other multipurpose tree foliage. Beyond its protein content, LGP was a rich source of phytonutrients, containing 6.8% CT and 2.4% flavonoids. These polyphenolic compounds, particularly tannins, play an important role in ruminal protein utilization. Zhang *et al.* [24] demonstrated that, under normal ruminal pH conditions, these active components bind with dietary proteins to form stable complexes, thereby reducing excessive microbial protein degradation in the rumen.

### Feed intake and nutrient digestibility

Dietary chemical composition, physical characteristics, and other related factors typically influence DM intake, which subsequently affects livestock productivity. In this study, no significant differences in feed intake were observed among treatment groups, indicating that LGP supplementation maintained normal feed consumption. This sustained intake may be attributed to the synergistic formulation of LGP, which likely counteracted the potential negative effects commonly associated with high-tannin diets. While CT from *L. leucocephala* contributes to dietary protein protection, ginger provides bioactive compounds, including gingerols and shogaols, that stimulate digestive enzyme activity. This combination may enhance diet palatability and reduce the intake-depressing effects commonly associated with single-source high-tannin supplementation.

Throughout the *in vivo* trial, no clinical signs of mimosine toxicity, such as alopecia, excessive salivation, depressed appetite, or visible goiter, were observed in any experimental animal. This absence of clinical toxicity supports the safety of the formulated pellet and the thermo-mechanical processing approach used in the study. Furthermore, the significant improvements in DM and neutral detergent fiber digestibility may be mechanistically attributed to the combined effects of CT and ginger bioactives. The targeted suppression of rumen protozoa by CT and flavonoids in LGP likely reduced bacterial predation, allowing greater proliferation of cellulolytic and fibrolytic bacteria. Consequently, enhanced bacterial activity, together with stimulation of salivary secretion and microbial enzymatic activity by ginger bioactives, may have improved structural carbohydrate degradation and overall nutrient digestibility.

These results are consistent with those of Phesatcha *et al.* [25], who reported an increase in total DM intake after supplementation with *Flemingia macrophylla* pellets. Similarly, Totakul *et al.* [26] showed that crossbred bulls did not alter feed consumption when supplemented with *Cnidoscolusa* *conitifolius* leaf pellets. Increasing LGP supplementation levels improved palatability and influenced rumen fermentation. Improvements in feed conversion ratio, growth rate, nutrient digestibility, and palatability have also been reported when plants, particularly ginger, were used to stimulate feed intake [27]. In the present study, LGP enhanced DM and neutral detergent fiber digestibility, likely because reduced protozoal populations decreased predation on fibrolytic bacteria. The bioactive compounds naturally present in ginger may also enhance animal health and growth by stimulating gastric secretions and digestive enzyme activity, thereby supporting feed utilization and production.

The addition of CT and SP may negatively affect nutrient digestibility, particularly protein digestion, when included at excessive levels. However, a moderate reduction in ruminal protein degradation can be beneficial if tannin-CP complexes dissociate in the lower digestive tract, thereby allowing subsequent protein digestion. Matra *et al.* [28] reported improved CP digestibility in dairy cows fed dragon fruit peel pellets. Although significant increases in DM and neutral detergent fiber digestibility were observed in this study (p < 0.05), the relatively high standard error, particularly for DM digestibility, should be noted. Such variability is common in *in vivo* studies using low-quality basal roughages, such as rice straw, and reflects inherent individual variation among animals within a small Latin square design. Nevertheless, the significant treatment effect indicates that the synergistic action of phytogenic compounds in LGP was sufficient to improve rumen microbial activity and feed degradation.

### Rumen fermentation characteristics, blood metabolites, and microbial populations

Rumen fermentation, microbial growth, and microbial activity were maintained within a pH range of 6.7-6.8. Improved rumen fermentation may be supported by maintaining ruminal pH through the strategic inclusion of feedstuffs containing phenolic acids [29]. Increasing LGP supplementation did not significantly alter NH_3_-N concentrations, which remained within the normal range of 13.7-18.5 mg/dL. BUN concentrations were closely associated with ruminal NH_3_-N concentrations, although no significant differences in BUN were observed among treatments. Phesatcha *et al.* [30] reported that CT improved nutrient utilization by forming protein-tannin complexes, decreasing ruminal feed protein degradation, and reducing ammonia production. In contrast, Matra *et al.* [28] found that ruminal NH_3_-N increased when Holstein crossbred bulls received dragon fruit peel pellets at 400 g/animal/day. In the present study, BUN concentrations ranged from 10.3 to 12.0 mg/dL and did not differ significantly among treatments, suggesting efficient use and absorption of available ruminal NH_3_-N for microbial synthesis. Similarly, Phesatcha *et al.* [25] reported normal BUN concentrations when tree leaf pellets were added to the diet as a CT source.

Patra and Saxena [31] reported that bioactive compounds can alter propionate formation when hydrogen is available in excess. Hydrogen is a major substrate for methanogenesis, whereas its use in propionate formation provides an alternative hydrogen sink. Therefore, inhibition of acetogenic bacteria and redirection of hydrogen toward propionate synthesis represent potential biological roles of tannins. A lower acetate-to-propionate ratio is generally associated with increased propionate concentration. Several studies have demonstrated that leguminous fodder shrubs can alter VFA profiles. Totakul *et al.* [26] reported that supplementation with *Cnidoscolus* leaf pellets increased propionate concentration and decreased the acetate-to-propionate ratio. Similar findings were reported by Phesatcha *et al.* [30], who observed increased propionate and reduced acetate proportions after leaf pellet supplementation.

Diets supplemented with CT and SP enhanced rumen fermentation, reduced CH_4_ production, decreased protozoal populations, and increased propionate production. Propionate concentration often increases when rumen methanogenesis is suppressed, as observed in this study. The shift in the VFA profile from acetate-to-propionate, along with reduced CH4 formation and redirected hydrogen utilization, may improve the energy availability to the host animal. These results are consistent with recent *in vitro* studies showing that *Z. officinale* mitigates CH_4_ emissions by altering rumen microbial activity [10]. Moreover, the synergistic use of phytogenic compounds has been shown to modify *in vitro* fermentation parameters and translate into reduced *in vivo* CH_4_ emissions while maintaining performance in dairy and beef cattle [32]. Similarly, phytogenic-based additives can suppress methanogenesis by shifting fermentation pathways toward propionate production [2].

The observed shift in rumen fermentation, particularly increased propionate and decreased CH_4_ production, is closely associated with the chemical properties of the phytogenic compounds. Rumen protozoa have a symbiotic relationship with methanogenic archaea, providing them with a habitat and hydrogen. The antiprotozoal actions of CT and flavonoids may disrupt this symbiosis and impair methanogenesis. Concurrently, to maintain thermodynamic balance in the rumen ecosystem, metabolic hydrogen may be redirected toward propionate synthesis. This competitive hydrogen sink can reduce CH_4_ emissions while providing more glucogenic energy to the host animal. Montoya-Flores *et al.* [8] reported a 14% reduction in CH_4_ production when *Leucaena* was included at a comparable level, which was attributed to the bioactive compounds present in *Leucaena*. Although formal correlation analysis between protozoal populations and CH_4_ production was not performed because CH_4_ was estimated indirectly using stoichiometric equations, the concurrent reduction in both parameters is consistent with established biological mechanisms. This parallel decline supports disruption of the symbiotic relationship between rumen ciliate protozoa and methanogenic archaea and further demonstrates the efficacy of LGP in favorably modifying the rumen microbiome.

Although LGP contained a relatively high CT concentration of 6.8%, the low daily supplementation rate, with a maximum of 150 g/head/day, ensured that total dietary CT remained below the threshold generally associated with impaired protein digestibility and well below commonly recommended safe inclusion limits. In addition, cattle in tropical regions, including Thai native beef cattle, may possess natural rumen adaptation. Their rumen microbiome often harbors mimosine-degrading bacteria, such as *Synergistes* *jonesii*, which can detoxify mimosine and its goitrogenic metabolites, including 3,4-dihydroxypyridine and 2,3-dihydroxypyridine. Thus, the formulation likely leveraged the beneficial effects of phytogenic compounds without approaching the threshold for mimosine-induced toxicity.

Essential oils in ginger may influence feed digestion and rumen fermentation. Saponins, which are among the bioactive constituents of ginger, may increase beneficial bacteria while decreasing protozoal populations. Higher LGP supplementation increased bacterial populations and reduced protozoal populations. Phesatcha *et al.* [30] similarly reported decreased protozoal and ruminal methanogen populations when beef cattle were supplemented with *Mitragyna* leaf pellets. The reduction of protozoa is a recognized mechanism by which bioactive compounds in both *Leucaena* and ginger suppress methanogenic archaea associated with protozoa, thereby improving nitrogen utilization and post-ruminal protein flow [5].

The reduction in protozoal populations, suppression of methanogenesis, and concurrent enhancement of microbial protein synthesis observed with LGP supplementation suggest a biological synergy among its constituent bioactive compounds. *Leucaena* is rich in CT, which can bind to the cell coat of ciliate protozoa and dietary proteins, impair cell membrane permeability, and inhibit enzyme activity. In parallel, the phytochemical profile of ginger, including flavonoids, gingerols, shogaols, and saponins, may complement this effect by disrupting lipid bilayers of methanogenic and protozoal cell membranes. This combination may create a dual-action inhibitory effect on cellular integrity, resulting in a more pronounced defaunation effect and greater bacterial proliferation and microbial protein synthesis than either ingredient alone. The rich phytochemical profile of these combined feedstuffs may therefore optimize fermentation kinetics and support higher microbial protein synthesis [4].

Ebeid *et al.* [33] reported that ginger supplementation improved digestion by increasing cellulolytic bacterial populations and enhancing salivary secretion, thereby increasing the secretion and activity of digestive enzymes. Ginger may benefit gastrointestinal ecology, improve feed stability, and limit the growth of pathogenic microorganisms, although excessive antimicrobial activity can reduce diet fermentability. Plant secondary metabolites and antiprotozoal actions of ginger components may therefore contribute to reduced total protozoal populations. Norrapoke and Pongjongmit [34] also reported that Mahad leaf pellet supplementation containing 15.6% CT significantly decreased protozoal populations in beef cattle.

The overall efficacy of LGP appears to be closely linked to dose-dependent synergy. At the optimal supplementation level of 150 g/head/day, which provided approximately 112.5 g of *Leucaena* leaves and 22.5 g of ginger powder, this study observed favorable ruminal outcomes. This dose increased propionate concentration and microbial protein synthesis while reducing protozoal populations and enteric CH_4_ emissions. Importantly, these effects occurred without adverse outcomes commonly associated with high-tannin interventions, such as reduced feed intake or compromised ruminal pH. Therefore, 150 g/head/day may represent a practical biological threshold at which complementary bioactives maximize fermentation efficiency while maintaining ruminal homeostasis.

### Microbial protein synthesis

Nitrogen intake increased with higher LGP supplementation, likely due to protein complex formation. Hung *et al.* [35] demonstrated that microbial nitrogen synthesis efficiency was enhanced by supplementation with *Leucaena* leaf pellets containing 24% CP. Rumen bypass protein also improved when dietary protein and CT levels increased with LGP supplementation. Rumen microorganisms play a central role in protein production, and changes in microbial growth directly influence amino acid availability. In the present study, LGP significantly increased microbial protein synthesis and the efficiency of microbial nitrogen synthesis.

The enhancement of microbial protein synthesis and microbial nitrogen synthesis efficiency can be explained by two major mechanisms. First, reversible binding of CT to dietary proteins at normal ruminal pH forms stable CT-protein complexes. This process protects protein from rapid ruminal degradation and synchronizes nitrogen release with carbohydrate fermentation for optimal microbial growth. Second, reduced protozoal predation on bacteria increases the net flow of intact microbial cells from the rumen to the lower gut. Together, these mechanisms improve the overall efficiency of microbial nitrogen synthesis. Viennasay and Wanapat [36] reported that *Flemingia* supplementation enhanced microbial nitrogen synthesis and its efficiency in lactating dairy cows, whereas Phesatcha *et al.* [37] found that supplementing beef cattle with pellets containing insect protein and phytonutrient plants improved microbial nitrogen synthesis efficiency.

Beyond the direct suppression of methanogenesis, the synergy between ginger bioactives and *Leucaena* CT may involve specific physical and enzymatic interactions related to CT-protein complex formation. While appropriate CT levels protect dietary protein from ruminal degradation, excessive CT can irreversibly bind proteins, reducing lower-gut digestibility. Ginger bioactives may counteract this risk through two mechanisms. First, gingerols can stimulate salivary secretion, and saliva contains proline-rich proteins that have a high affinity for tannins. Increased salivary flow may therefore buffer excess CT and prevent excessive inhibition of dietary protein digestion in the rumen. Second, after reversible CT-protein complexes dissociate in the acidic environment of the abomasum, gingerols may stimulate lower-gut protease secretion, improving breakdown and absorption of liberated amino acids. This dual-action mechanism may explain the increased microbial nitrogen flow and sustained nutrient digestibility observed in LGP-supplemented cattle.

### Practical relevance, limitations, and future perspectives

The synergistic efficacy of LGP should be interpreted in relation to previous studies that evaluated these phytogenic sources independently. Several previous *in vivo* studies using *Leucaena* alone reported a risk of reduced DM and structural carbohydrate digestibility at high inclusion levels due to excessive CT-protein binding [8]. In contrast, the present study demonstrated significantly improved digestibility of DM and neutral detergent fiber, suggesting that ginger bioactives in LGP may have counteracted potential CT-induced microbial inhibition. Similarly, although *in vitro* studies have documented the antimethanogenic and antimicrobial effects of ginger extracts, their practical *in vivo* application may be limited by the rapid ruminal degradation of volatile compounds. In contrast, the pelleted LGP matrix may help stabilize these bioactives and achieve a marked reduction in protozoal populations, along with increased microbial protein synthesis, at a conservative supplementation level of 150 g/head/day. Therefore, the combined formulation appears to provide greater rumen-modulatory benefits than either *Leucaena* or ginger alone.

From a practical perspective, LGP functions as a ready-to-use technology for resource-limited tropical farmers. Its reliance on standard pelleting equipment and locally available ingredients suggests strong potential for cooperative-level scale-up and broader commercialization. By using locally abundant materials from Roi Et Province and evaluating their efficacy in Thai native beef cattle fed a low-quality rice straw-based diet, this formulation directly addresses practical nutritional constraints in tropical smallholder systems. The innovation of this study extends beyond combining ingredients; it includes the development of a specific pelleted matrix. Unlike most previous studies that evaluated *Leucaena* as loose leaves or unprocessed meal, this study used cassava chips and molasses as binders, together with mechanical processing and sun drying. This thermo-mechanical approach may improve pellet integrity, storage stability, and ease of feeding, while potentially enhancing the bioavailability of volatile gingerols and further reducing residual mimosine within the pellet matrix.

Mimosine toxicity remains a recognized limitation in *Leucaena* utilization, but LGP may provide a dual mitigation strategy. In addition to physical mimosine reduction through drying and pelleting, enhanced fermentation and bacterial proliferation stimulated by ginger bioactives may further support ruminal detoxification of residual mimosine and its secondary metabolites. Consequently, this phytogenic formulation may promote sustainable and adoptable feeding practices in tropical regions.

The CT concentration must also be interpreted in relation to total dietary intake. Although LGP contained 6.8% CT, the maximum supplementation rate of 150 g/head/day diluted the net CT contribution to only a small fraction of total dietary DM. This level is likely within a beneficial range, allowing CT to act as a phytogenic rumen modifier without reaching concentrations that negatively affect feed intake or nutrient digestibility.

Despite these promising findings, several limitations should be acknowledged. First, the 4 × 4 Latin square design, although appropriate for evaluating ruminal fermentation, included only four animals, short experimental periods of 21 days, and exclusively female cattle. These factors limit direct extrapolation to long-term production outcomes, including average daily gain, final BW, carcass traits, and responses in male cattle. Second, although the physical integrity of LGP was adequate for handling and daily feeding, formal evaluation of pellet characteristics, including hardness, durability, and water stability, was not performed. Third, residual mimosine and specific ginger bioactives in the final pellet were not quantified because of analytical constraints. Finally, classical methods such as hemocytometer counting and VFA-based stoichiometric estimation do not identify specific microbial taxa or measure absolute CH_4_ emissions.

Future studies should therefore include long-term growth trials to validate practical application and economic benefits. Direct quantification of CH_4_ using respiration chambers or related technologies, comprehensive mimosine adaptation studies, quantification of residual mimosine and ginger bioactives, and advanced molecular microbiome analysis are also recommended to clarify the long-term physiological, microbial, environmental, and economic impacts of this synergistic pellet.

## CONCLUSION

Supplementation with LGP at 150 g/head/day, representing approximately 2.5% of total dietary DM, improved rumen fermentation efficiency in Thai native beef cattle by increasing DM and neutral detergent fiber digestibility, enhancing propionate production, increasing bacterial populations, and improving microbial protein synthesis. In addition, LGP reduced protozoal populations and estimated CH_4_ production without affecting feed intake, ruminal pH, NH_3_-N, or BUN concentrations. No clinical signs of mimosine toxicity were observed, indicating that the pelleted formulation and controlled supplementation level were well tolerated.

These findings suggest that LGP is a practical phytogenic feed additive for tropical beef production systems, particularly for cattle maintained on low-quality rice straw-based diets. The major strength of this study is the development of a locally available, low-cost, and easy-to-produce pelleted formulation that combines the complementary bioactive effects of *L. leucocephala* and ginger.

However, the study was limited by its short experimental duration, small sample size, inclusion of only female cattle, indirect estimation of CH_4_ production, and lack of quantification of residual mimosine and ginger bioactive compounds. Future long-term *in vivo* studies should include direct CH_4_ measurements, evaluation of growth and carcass performance, mimosine safety assessment, pellet quality testing, and molecular analysis of the rumen microbiota.

Overall, LGP offers a promising, scalable, and environmentally sustainable strategy to improve rumen function, enhance microbial protein synthesis, and reduce enteric CH_4_ emissions in tropical beef cattle production.

## DATA AVAILABILITY

The supplementary data can be made available from the corresponding author upon request.

## GENERATIVE AI DECLARATION

The authors declare that no generative artificial intelligence or AI-assisted technologies were used in the writing, analysis, or preparation of this manuscript.

## AUTHOR’S CONTRIBUTIONS

**PW and BP:** Conceptualization, study design, manuscript drafting, and revision. PW, MM, and KP: Methodology and data collection and analysis. PW, MM, and TA: Formal analysis and investigation. PW: Statistical analysis. All authors critically revised the manuscript, read, and approved the final version.

## References

[1] Lillehoj H, Liu Y, Calsamiglia S, Fernandez-Miyakawa ME, Chi F, Cravens RL (2018). Phytochemicals as antibiotic alternatives to promote growth and enhance host health. Vet Res.

[2] El-Zaiat HM, Masood HA, Al Hinai SS, Al-Shuraiqi NM, Al-Hashimi HA, Al-Nabhani AM (2025). Assessment of different phytogenic-based additives on in vitro rumen fermentation profile and methane emissions. Front Vet Sci.

[3] Singsai K, Sakdavirote A, Wechpanichkitkul K, Moonsamai A (2020). The comparison of phenolic compounds, flavonoids and antioxidant activities of the ethanolic extracts of shoots, leaves, fruits and seeds of Leucaena leucocephala. Naresuan Phayao J.

[4] Ammar H, Kholif AE, Henrique Horst E (2024). Chemical composition and in vitro rumen fermentation kinetics of leaves and stems of Moringa oleifera and Leucaena leucocephala as potential feedstuffs for sheep. Cogent Food Agric.

[5] Gaviria-Uribe X, Barahona R, Ruiz-Cortés ZT, Bolivar-Vergara DM (2022). The effects of two species of Leucaena on in vitro rumen fermentation, methane production and post-ruminal protein supply in diets based on Urochloa hybrid cv. Cayman. Agronomy.

[6] De Angelis A, Gasco L, Parisi G, Danieli PP (2021). A multipurpose leguminous plant for the Mediterranean countries: Leucaena leucocephala as an alternative protein source in animal nutrition. Animals.

[7] Melesse A, Steingass H, Schollenberger M, Holstein J, Rodehutscord M (2019). Nutrient compositions and in vitro methane production profiles of leaves and whole pods of twelve tropical multipurpose tree species cultivated in Ethiopia. Agrofor Syst.

[8] Montoya-Flores MD, Molina-Botero IC, Arango J, Romano-Muñoz JL, Solorio-Sánchez FJ, Aguilar-Pérez CF (2020). Effect of dried leaves of Leucaena leucocephala on rumen fermentation, rumen microbial population, and enteric methane production in crossbred heifers. Animals.

[9] Aoetpah A, Oematan JS, Manafe ME, Sun W, Banunaek MF, Wirawan IGK (2024). Types of tropical legume leaf meal in dietary concentrate increased the production of forage finished beef Bali cattle. JIIP.

[10] Balamurugan R, Kathirvelan C, Abinaya A (2025). Evaluating the methane mitigation potential of ginger rhizomes (Zingiber officinale) by in vitro study. J Vet Anim Sci.

[11] Srinivasan K (2017). Ginger rhizomes (Zingiber officinale): A spice with multiple health beneficial potentials. Pharma Nutrition.

[12] Kairalla MA, Aburas AA, Alshelmani M (2022). Effect of diet supplemented with graded levels of ginger (Zingiber officinale) powder on growth performance, hematological parameters, and serum lipids of broiler chickens. Arch Razi Inst.

[13] Al-dain QZS, Jarjeis EA (2015). Vital impact of using ginger roots powder as feed additive to the rations of local Friesian dairy cows and its effect on production & economic efficiency of milk and physiological of blood. Kufa J Vet Med Sci.

[14] Association of Official Analytical Chemists (AOAC) International (2012).

[15] Van Keulen J, Young BA (1977). Evaluation of acid-insoluble ash as a natural marker in ruminant digestibility studies. J Anim Sci.

[16] Van Soest PJ, Robertson JB, Lewis BA (1991). Methods for dietary fiber, neutral detergent fiber, and nonstarch polysaccharides in relation to animal nutrition. J Dairy Sci.

[17] Reabaya A, Belghith SI, Baghdikian B, Leddet VM, Mabrouki F, Olivier E (2015). Total phenolic, total flavonoid, tannin content, and antioxidant capacity of Halimium halimifolium (Cistaceae). J App Pharm Sci.

[18] Chen XB, Gomes MJ (1995). Estimation of microbial protein supply to sheep and cattle based on urinary excretion of purine derivatives: an overview of the technique details.

[19] Mathew S, Sagathevan S, Thomas J, Mathen G (1997). An HPLC method for estimation of volatile fatty acids in ruminal fluid. Indian J Anim Sci.

[20] Moss AR, Jouany JP, Newbold J (2000). Methane production by ruminants: its contribution to global warming. Ann Zootech.

[21] Galyen M (1989). Laboratory procedure in animal nutrition research.

[22] Crocker CL (1967). Rapid determination of urea nitrogen in serum or plasma without deproteinization. Am J Med Technol.

[23] Steel RGD, Torrie JH, Dickey DA (1997).

[24] Zhang J, Zu X, Cao Z (2019). Effects of different tannin sources on nutrient intake, digestibility, performance, nitrogen utilization, and blood parameters in dairy cows. Animals.

[25] Phesatcha B, Phesatcha K, Viennaxay B, Thao NT, Wanapat M (2021). Feed intake and nutrient digestibility, rumen fermentation profiles, milk yield and compositions of lactating dairy cows supplemented by Flemingia macrophylla pellet. Trop Anim Sci J.

[26] Totakul P, Viennasay B, Sommai S, Matra M, Infascelli F, Wanapat M (2021). Supplemental effect of Chaya (Cnidoscolus aconitifolius) leaf pellet on rumen fermentation, nutrients digestibility and microbial protein synthesis in growing crossbred bulls. Ital J Anim Sci.

[27] Wang LM, Mandell IB, Bohrer BM (2020). Effects of feeding essential oils and benzoic acid to replace antibiotics on finishing beef cattle growth, carcass characteristics, and sensory attributes. Appl Anim Sci.

[28] Matra M, Totakul P, Viennasay B, Phesatcha B, Wanapat M (2021). Dragon fruit (Hylocereus undatus) peel pellet as a rumen enhancer in Holstein crossbred bulls. Anim Biosci.

[29] Viennasay B, Totakul P, Matra M, Phesatcha B, Wanapat M (2022). Influence of bamboo grass (Tiliacora triandra, Diels) pellet supplementation on in vitro fermentation and methane mitigation. J Sci Food Agric.

[30] Phesatcha B, Phesatcha K, Wanapat M (2022). Mitragyna speciosa Korth leaf pellet supplementation on feed intake, nutrient digestibility, rumen fermentation, microbial protein synthesis and protozoal population in Thai native beef cattle. Animals.

[31] Patra AK, Kamra DN, Agarwal N (2010). Effects of extracts of spices on rumen methanogenesis, enzyme activities and fermentation of feeds in vitro. J Sci Food Agric.

[32] Khelil-Arfa H, Tondini SM, Belanche A (2025). Effect of a Combination of Phytogenic Compounds on In Vitro Rumen Fermentation Parameters and In Vivo Lactation Performance and Methane Emissions in Dairy Cows. Methane.

[33] Ebeid HM, Mengwei L, Kholif AE (2020). Moringa oleifera Oil Modulates Rumen Microflora to Mediate In Vitro Fermentation Kinetics and Methanogenesis in Total Mix Rations. Curr Microbiol.

[34] Norrapoke T, Pongjongmit T (2025). The use of Mahad leaf pellet feed affects the fermentation process and rumen micro-biota in beef cattle. CABI Agric Biosci.

[35] Hung LV, Wanapat M, Cherdthong A (2013). Effect of Leucaena leaf pellet on bacterial diversity and microbial protein synthesis in swamp buffalo fed on rice straw. Livest Sci.

[36] Viennasay B, Wanapat M (2020). Enhancing lactating dairy cow rumen fermentation and production with Flemingia silage containing phytonutrients. Livest Sci.

[37] Phesatcha B, Phesatcha K, Matra M, Ampapon T, Wanapat M (2026). Potential use of insect protein and phytonutrient-based tropical plant supplementation on rumen fermentation characteristics and microbial protein synthesis in Thai native beef cattle. Anim Biosci.

